# ‘I’m sick of being the problem’: Autistic mothers’ experiences of interacting with schools for their autistic children

**DOI:** 10.1177/13623613241297223

**Published:** 2024-11-24

**Authors:** Aspasia Stacey Rabba, Jodie Smith, Gabrielle Hall, Vanessa Alexander, Kate Batty, Poulomee Datta, Emma Goodall, Melanie Heyworth, Siobhan Lamb, Wenn Lawson, Rozanna Lilley, Katy Reid, Najeeba Syeda, Elizabeth Pellicano

**Affiliations:** 1 Macquarie University, Australia; 2 Monash University, Australia; 3 La Trobe University, Australia; 4 University of Technology Sydney, Australia; 5 Positive Partnerships, Australia; 6 Reframing Autism, Australia; 7 University College London, UK

**Keywords:** autistic parents, autistic students, parent–teacher partnerships, participatory research, school experiences

## Abstract

Successful partnerships between families and schools can substantially improve children’s success in and out of school. Yet parents of autistic children often report considerable challenges with their children’s education. Almost no research, however, has examined autistic parents’ own experiences. We, therefore, sought the views and experiences of autistic parents’ interactions with schools for their autistic children. We conducted semi-structured interviews with 31 autistic mothers of autistic children engaged in education in Australia. We analysed the data using reflexive thematic analysis. Autistic mothers repeatedly expressed feeling like they were in a ‘war zone’, fighting ongoing battles to gain the necessary support for their autistic child and were ‘sick of being [viewed as] the problem’. They also felt that their expertise as parents – and experiential expertise as autistic parents – was often overlooked or ignored. All of this had negative effects on their and their children’s mental health. Despite these negative experiences, autistic mothers who had experienced positive interactions with schools highlighted the importance of mutual respect in establishing and maintaining successful school partnerships and how their own determination and self-advocacy helped to foster these relationships. This research sheds light on the critical ingredients of effective home–school partnerships for autistic families.

**Lay abstract:**

Good relationships between families and schools make a difference to children’s learning – and the same goes for autistic children. But parents of autistic children often find it very stressful interacting with teachers and school staff. In this study, we focused on autistic parents of autistic children. We wanted to know about their experiences of interacting with schools and the impact these had on them and their children. We spoke to 31 autistic mothers of autistic children about their experiences. They told us that they felt they were constantly fighting with schools to get the support needed for their autistic children and compared it to like being in a ‘war zone’. They were ‘sick of being [viewed as] the problem’ and felt that their views and autistic expertise were not taken seriously by teachers and schools. This was damaging to their autistic children’s mental health as well as their own. Autistic mothers did share some positive experiences too. They spoke about the value of mutual respect and its impact on successful school partnerships. Autistic mothers also spoke about standing up for themselves and their children and how this advocacy and self-advocacy helped them to build better relationships with schools. This research showed how difficult it can be for autistic families to interact with teachers and schools and the impact this can have on the whole family. It also showed us that strong, trusting relationships between school and families are possible – when autistic parents feel safe, and when their knowledge and lived experience are taken seriously by educators.

Effective partnerships between families and schools are important for any child (Christenson, 2004; Lilley, 2019) and can have a substantial impact on children’s success in school (Sheridan et al., 2012). These partnerships can be especially important for autistic children (Azad & Mandell, 2016; Azad et al., 2016; Garbacz et al., 2016; Lilley, 2019), who may face challenges with stigma and rejection, and increased risks of poor academic and mental health outcomes (Ashburner et al., 2019; Aubé et al., 2021; Feldman et al., 2022; Shkedy et al., 2020; Simonoff et al., 2012; Sreckovic et al., 2014). Yet, parents of autistic children often report substantial challenges navigating their children’s education, repeatedly highlighting the lack of understanding and access to autism-specific knowledge, expertise and support for their children; poor communication channels; adversarial relationships with teachers; and the lack of two-way partnerships with schools (Kendall & Taylor, 2016; Lilley, 2019; McNerney et al., 2015). Despite confidence that they know their autistic children best, parents often describe not being listened to as well as being excluded from classrooms, resulting in them feeling isolated and unsupported (Lilley, 2015; Makin et al., 2017). Yet, almost all existing research has focused on non-autistic parents’ experiences of interacting with schools.^1^ Here, we focus on autistic parents of autistic children to understand their views and experiences.

There is remarkably little research with autistic parents of autistic children – in part because, historically, autistic parents were thought to be rare (Ritvo et al., 1994). The broadening of diagnostic criteria for autism (American Psychiatric Association, 2013), and increase in the availability of diagnostic services, has led to a rise in adult diagnoses, with more parents of autistic children being diagnosed, sometimes following their child’s diagnosis (Crane et al., 2021; Huang et al., 2020; Lilley et al., 2022). In addition, there has also been a significant rise in parents self-identifying as autistic, with frequent reports of parents exploring their own autistic identity outside of formal diagnostic processes as a result of their increased understanding of, and resonance with, their child’s autistic identity, needs and challenges (Harmens et al., 2022).

Emerging research suggests that parenting can be a rewarding experience for autistic parents – one full of love and joy (Dugdale et al., 2021; Heyworth et al., 2021). Autistic parents can also share an intense connection with their children, in part because of their experiential expertise and shared identity, or unique knowledge and insight of being autistic (Crane et al., 2021; Dugdale et al., 2021; Heyworth et al., 2021; Lilley et al., 2023). Yet, parenting can also be challenging (Hampton et al., 2022; McDonnell & DeLucia, 2021; Pohl et al., 2020). The additional demands that come with parenting (e.g. domestic chores, transporting children to and from schools and extra-curricular activities, organising playdates with peers) can be particularly overwhelming for autistic parents (Heyworth et al., 2021; Pohl et al., 2020). Interactions with professionals, including teachers, can also be a source of anxiety. Autistic parents find it more difficult than non-autistic parents to communicate with non-autistic professionals about their autistic child and can feel misunderstood during those interactions (Pohl et al., 2020). Furthermore, they also report being reluctant to disclose their autism diagnosis, for fear of being judged as a ‘bad parent’ (Rogers et al., 2017). These challenges can take a toll on mental health, with autistic mothers reporting they often have few formal supports on which to draw (Heyworth et al., 2021).

Interactions between autistic parents and non-autistic professionals, including teachers, might be particularly challenging because of fundamental differences in the way each group experiences the world (Milton, 2012). While it has long been thought that autistic people struggle to understand others’ thoughts and feelings, it is now widely recognised that non-autistic people can also struggle to work out the minds of autistic people. These struggles reveal a ‘double empathy problem’ (Milton, 2012), which can lead to communication breakdowns between autistic and non-autistic people (Crompton et al., 2020). This issue is important given that effective communication is critical to facilitating successful home–school partnerships between (non-autistic) parents of autistic children and their children’s teachers (Azad & Mandell, 2016). Yet we know virtually nothing about the extent and nature of these interactions for autistic parents.

## This study

This study sought to redress the imbalance in this research, by eliciting the views and experiences of autistic parents’ school interactions about their autistic children. To achieve this, we – a team of autistic researchers and community members and non-autistic researchers – conducted in-depth semi-structured interviews, focusing on the nature and impact of these interactions on the autistic parent themselves, their children and family.

## Method

### Participants

We recruited autistic parents through purposive and snowball sampling, initially through social media and community connections. People were eligible if they were ⩾ 18 years, were diagnosed or self-identified as autistic, and had at least one child with a clinical diagnosis of autism engaged in education in Australia.

Thirty-one autistic mothers met these criteria (see Table 1).^2^ They ranged from 34 to 59 years (M = 44.6 years), and were either diagnosed (n = 25) or self-identified as autistic (n = 6) later in life (M age = 41 years). Most were university educated (n = 20; 64%) and employed in some way (n = 21; 68%). Almost all were born in Australia and most identified as being of white ethnic background. One mother (3%) identified as Aboriginal-Australian and one as Indian-Australian (3%). Most reported additional mental and physical health diagnoses, most commonly depression, anxiety and gastrointestinal issues.

**Table 1.** Demographics of autistic parents (n = 31) involved in the study.

	N (%) or M (SD), range
Parent characteristics	
Gender	
Woman	31 (100%)
Age	44.6 (6.1), 34–59
Average age at diagnosis (years)^a^	41.2 (5.4), 34–50
Number of autistic children	
1	10 (32.3%)
2	15 (48.4%)
3	5 (16.1%)
4	1 (3.2%)
Total number of children (mode)	2 (1.9), 1–6
Parents’ diagnosed conditions	
Mental health/Neurodevelopmental conditions	
Depression	22 (71.0%)
Anxiety disorder	20 (64.5%)
Post-traumatic stress disorder	7 (22.6%)
ADHD/ADD	6 (19.4%)
Eating disorder	6 (19.4%)
Physical health conditions	
Gastrointestinal issues	12 (38.7%)
Chronic pain	8 (25.8%)
Sleep issues	8 (25.8%)
Obesity	7 (22.6%)
Autoimmune condition	7 (22.6%)
Employment status	
Part-time employment	10 (32.3%)
Self-employed	7 (22.6%)
Homemaker/full-time parent	7 (22.6%)
Full-time employment	4 (12.9%)
Unable to work due to disability	2 (6.5%)
Studying full-time	1 (3.2%)
Education level	
University Degree	20 (64.5%)
TAFE Certificate/Diploma	8 (25.8%)
Trade/Technical Certificate	1 (3.2%)
Completed High School	1 (3.2%)
Started High School	1 (3.2%)
NDIS^b^ plan in place	
No	21 (67.7%)
Yes	9 (29%)
Pending approval	1 (3.2%)
Household income	
US$1 to US$25,000 per year (US$1–US$381 per week)	6 (19.4%)
US$25,001 to US$50,000 per year (US$482–US$962 per week)	7 (22.6%)
US$50,001 to US$78,000 per year (US$963–US$1500 per week)	3 (9.7%)
US$78,001 to US$104,000 per year (US$1501–US$2000 per week)	6 (19.4%)
US$104,001 or more per year (more than US$2001 per week)	2 (6.5%)
Prefer not to say	7 (22.6%)
Ethnic background	
White ethnic background	20 (64.4%)
Mixed: Aboriginal-Australian	1 (3.25%
Mixed: Indian-Australian	1 (3.25%)
Prefer not to say	9 (29.1%)

ADHD: attention-deficit hyperactivity disorder; ADD: attention-deficit disorder; TAFE: technical and further education; NDIS: National Disability Insurance Scheme.

a n = 25 had received a clinical diagnosis of an autism spectrum condition. The remaining six mothers reported a self-diagnosis.

b The NDIS, funded by the Australian Government, provides no-fault insurance cover for Australians (aged < 65 years), who are born with or acquire a permanent and significant disability. It provides disability funding for support and services directly to individuals and is designed to give them more choice and control over their care. Those not in receipt of the NDIS funding accessed services and supports through other means, including government, charitable and private provision.

Together, these mothers were caregivers to 59 autistic children, ranging from 5 to 30 years (see Table 2; one mother reported having two autistic children: one school aged and one aged 30 years). Most had more than one autistic child. Children ranged in education level from preschool (n = 5; 8.5%), primary school (n = 13; 22.0%) through to secondary school (n = 16; 27.1%), with many students also engaged in home-schooling (n = 21; 35.6%), who typically did not identify with a specific year level.

**Table 2.** Demographics of autistic children of the autistic parents.

	N (%) or M (SD), range
Child characteristics	
Gender^a^	
Boy	30
Girl	23
Non-binary	3
Prefer not to say	3
Age	12 (4.4), 5–30
NDIS plan in place	
Yes	46 (78%)
No	13 (22%)
Type of education setting	
Home-schooling	26 (44.0%)
Mainstream – with extra support	21 (35.6%)
Mainstream – with no extra support	7 (11.9%)
Virtual school/online school	3 (5.1%)
General special school	1 (1.7%)
Other	1 (1.7%)
Enrolment type	
Preschool	5 (8.5%)
Primary school	13 (22.0%)
Secondary school	16 (27.1%)
Home-schooling	21 (35.6%)
Did not respond	4 (6.8%)

NDIS: National Disability Insurance Scheme.

a n =59 had received a clinical diagnosis of an autism spectrum condition.

### Procedure

Ethical approval was granted by Macquarie University Human Research Ethics Committee (Ref No: 5202196412836) and all participants provided written informed consent before taking part.

Autistic mothers were initially asked to complete a brief (10 min) online survey, via Qualtrics, to elicit their and each of their children’s demographic, diagnostic and schooling information. Next, we conducted in-depth semi-structured interviews with autistic mothers (from June to September 2021), via Zoom, as COVID-19-related restrictions at the time prohibited face-to-face research. During the interviews, we sought to contextualise autistic parents’ experiences and characterise the multiple factors influencing them and their children – including their family, peers, educational setting, school community, societal attitudes, and broader systemic ideologies and values. We, therefore, asked open-ended questions about their family; their experiences of school; the degree and nature of communication with teachers; their participation in school activities; and their views on what an ideal home–school partnership might look like (see Appendix A for full interview schedule). We emailed the primary questions to participants ahead of the interview.

### Community involvement

We incorporated autistic experiential expertise at multiple levels (Fletcher-Watson et al., 2019; Nicolaidis et al., 2011; Pellicano & Stears, 2011) to ensure that our research was attentive to the realities of participants’ everyday lives and to enhance the effectiveness of the research itself (Pellicano et al., 2022). First, the research team comprised autistic scholars and advocates, who were also autistic parents of autistic children. All team members actively contributed from the project outset (as named grant applicants), making collaborative decisions regarding methodology, contributing to the analysis and interpretation of the findings and writing the article. Second, we employed an autistic team member (G.H.) to interview all our autistic parents. We had anticipated that some autistic parents’ experiences with schools may have been challenging, hence having an autistic interviewer served to increase the likelihood of our participants feeling heard and understood (Forsythe et al., 2019; Gibson & Abrams, 2003; Pellicano et al., 2022). Third, we established a community Advisory Group consisting of four autistic parents of autistic children, none of whom were researchers. These parents attended eight meetings across the project, providing their insights on the interview questions, the interpretation of the results, recommendations and dissemination pathways. Autistic parent advisors were paid for their time and expertise. All team members and advisory group members are authors of this article.

### Data analysis

**Figure 1.**
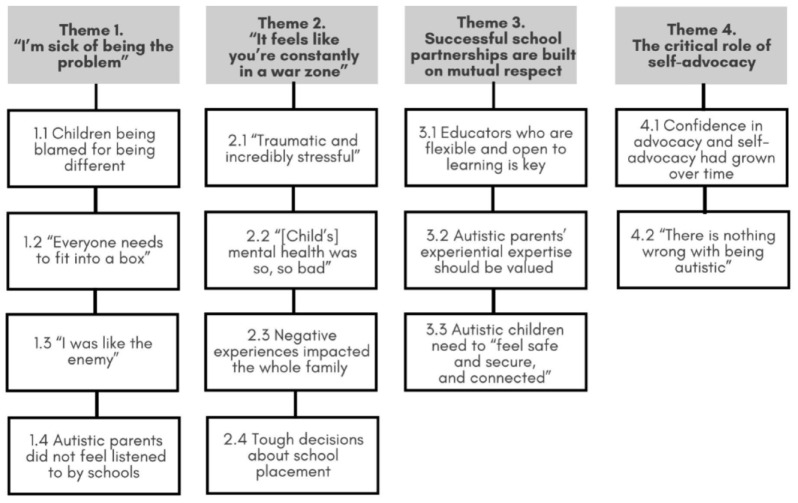
Themes and subthemes identified from our autistic parent participants’ interview responses.

Interviews ranged between 39 and 90 min (M = 68.0, SD = 14.1), recorded with participants’ prior permission and subsequently transcribed verbatim. Prior to analysis, we returned participants’ written transcripts to check for accuracy. We used reflexive thematic analysis (Braun and Clarke (2019) within an essentialist framework, in which we report the experiences, meanings and reality of the participants and provide a comprehensive understanding of complex, dynamic and multidimensional phenomena. Our analysis was informed by our collective experience and training in psychology (A.S.R., W.L. and E.P.), speech pathology (J.S.), education (A.S.R., V.A., K.B., P.D., E.G., M.H., S.L., R.L., K.R. and E.P.), anthropology (R.L.), nursing (G.H.) as well as positionalities as Autistic people (K.B., E.G., G.H., M.H., S.L., W.L. and K.R.) and parents of autistic children (K.B., P.D., G.H., M.H., S.L., W.L., R.L. and K.R.). Following transcription, the lead authors immersed themselves in the data, taking notes on striking and recurring observations. Codes were developed and applied to each transcript (managed in NVivo, v12) in discussion with the advisory group and research team. Next, a draft thematic map showing potential (sub)themes was generated, along with all relevant quotes, and revised during multiple discussions with the advisory group. Finally, the revised thematic map was reviewed by the broader team and members of our community advisory group, during which changes were made, especially editing and reframing theme names for emphasis, clarity and transparency, prior to being finalised (see Figure 1). Analysis was, therefore, iterative and reflexive, moving backward and forward between data and analysis.

## Results

We identified four themes (see Figure 1). Below, themes are highlighted in bold and subthemes are italicised; quotes are attributed via fictitious initials to maintain anonymity.

### Theme 1: ‘I’m sick of being the problem’

Overall, our autistic mothers reported consistently challenging interactions with schools. They repeatedly reported feeling perceived to be ‘the problem . . . even though we were so polite and so gentle and so proactive’ (LK). As one mother described: ‘I was a convenient excuse for every failure, that actually, somehow, was my failure’ (JR). These damaging perceptions began with their *children being blamed for being different* (subtheme 1.1): ‘even just in terms of him being able to cope with the day-to-day school life, it was always that he was a problem’ (ST). One autistic parent shared that her child’s teacher had explicitly said, ‘your child’s got problems’, which had a negative impact on her own and her children’s engagement with school: ‘I ended up pulling them out of school, just for the last three days of term, because I was like, I’m exhausted, they were exhausted’ (AY). Parents felt that these negative perceptions resulted from fundamental misunderstandings of autism: ‘I realised pretty quickly from the staff and the principal that that’s what they thought autism is – this extreme behaviour – and these are naughty kids’ (LK).

Autistic parents were well aware that ‘being an invisible neurodivergent person is not an easy path in the school environment’ (PQ). This was made more challenging due to what they felt were narrow views of education, where ‘*everyone needs to fit into a box* (subtheme 1.2) and get out the other end in a certain way’ (AY). These views were often influenced by their own negative school experiences as a child – experiences that contributed to their concerns for their children: ‘I went to a very academic school, that was very, very, very square peg, round hole, and there’s probably a layer of my worry . . . I feel like I can see it happening already’ (AY). They often described having little-to-no opportunity to express their needs at school, only to learn this later in life when their own diagnosis helped them to develop ‘a confidence that comes with self-acceptance’ (KC). They reported how their children often learned in different ways (‘half of his day, from my understanding, sitting under his desk, doing schoolwork’; NW), suggesting that teachers and school staff should have ‘to think outside that box, and work with what works for [child]’ (RB) – which unfortunately did not always happen (‘they just didn’t get her’; ML). Parents felt that their individual schools’ normative ‘mindset’ reflected ‘a broader problem’ (AY), an education system that focuses ‘on the outcome and the comparison, not the nourishing and development of a person’ (RB). Some described seeing this approach play out with other

‘parents trying to navigate school with autistic kids. They’re trying to make their kids fit in and trying to make their kids be normal. And I find it really hard to watch it and not be like, it doesn’t have to be like this’ (LK).

As one mother put it:

‘being autistic is just like having brown eyes, it’s just part of you, and it has benefits, and it has things that sometimes make things a little extra tricky for you; unfortunately, the world isn’t always set up to support that’. (AY)

Consequently, autistic mothers reported having to fight for schools to acknowledge ‘my child’s needs and advocate’ (RB) for the right support, but they often felt ‘*I was like the enemy*’ (subtheme 1.3). They reported feeling blamed for their child being autistic (‘that was always put back onto us and always put back onto our child’; LK) and, for some, they were explicitly held to be responsible: ‘My parenting was called into question’ (RB). They also spoke of being weighed down by the never-ending ‘battle’ with schools: ‘It’s not fair to ask parents to have to do that, and to feel they’re fighting, when we are, ourselves, such a vulnerable population’ (JR). This same parent explained, ‘advocacy doesn’t always have to be combative, it doesn’t always have to be that way, but it seems like school’s set us up to be a really aggressive kind of power struggle, and we [parents] don’t have the power’.

Ultimately, *autistic parents did not feel listened to by schools* (subtheme 1.4). Participants were perplexed that their parenting expertise and intimate knowledge of their own child were not appreciated or harnessed by teachers and other school staff:

‘it’s difficult when school staff only ever see you as a parent, you deliver the same message that a professional would deliver, but they don’t see any value in what you say because you’re there as a parent and not a professional’. (ST)

Moreover, they were particularly baffled when their experiential expertise – of being an autistic parent – was not valued, expressing that ‘I have a little bit more of insight than the average normal mother might do, with an autistic kid’ (GI). They felt that ‘teachers and principals feel that they know better than parents, and therefore that, as a parent, you have very little to offer . . . ultimately, your views and your insight and your experiential knowledge is ignored’ (JR).

### Theme 2: ‘It feels like you’re constantly in a war zone’

Despite being fierce advocates for their children, autistic parents’ negative interactions with schools took a toll on themselves, their children and their family. They repeatedly described how ‘*traumatic and incredibly stressful*’ it can be advocating for their child/ren (subtheme 2.1). Some reported that school staff made them ‘feel guilty for having to beg for the smallest accommodations’ (JR), while others recounted being ‘stonewalled . . . every time I would try and step over another hurdle of bureaucracy or funding . . . it was shaming and othering, and again, it makes you feel powerless and hopeless’ (TU). They described that the cumulative stress meant that their ‘own mental health has started suffering’ (LF): ‘I’m having full-blown heart palpitations at the thought of having to deal with teaching staff and school and the whole system’ (RB). One parent reported having ‘given up doing heavy advocacy about two years ago, because it burnt me out and it wasn’t making any difference’ (TU). Another parent spoke of feeling ‘constantly traumatised by not knowing whether people were going to look after my kids appropriately’ (LF). They felt failed by ‘the people that are supposed to be understanding and supportive . . . a school is supposed to be a community’ (RB). Another mother explained:

The judgement and ostracism and exclusion [from school] was just like the rest of my life. I wanted that not to be the case for my kids . . . I thought if I could do a better job, and again, I took that on as guilt, that I wasn’t doing a better job with the kids. (JR)

It was not surprising that parents recounted substantial impacts on their mental health, including ‘massive mental health issues’ (DE) and ‘several burnouts’ (TU).

The effects of these negative school interactions were far-reaching. One parent conveyed how their ‘*[child’s] mental health was so, so bad*’ (subtheme 2.2), with one child ‘talking about killing herself at eight’ (RB). Parents repeatedly spoke of ‘the bullying, the anxiety issues, a lot of mental health stuff’ (VJ). Another recounted how her autistic child ‘at one point told me that he’d rather die than go to school’ (GI). This same parent described the compassion she experienced when her son was suicidal, and how the goodwill stopped when he was labelled as ‘misbehaving’ by the school principal: ‘He had a kid that was constantly poking and kicking him. When he retaliated, he was grabbed by his shoulder, and hauled out of the line, by the principal’ (GI).

Unsurprisingly, these negative experiences further compounded autistic parents’ mental health and also *impacted the whole family* (subtheme 2.3). Some autistic parents reported that ‘our (family) relationship was very fractured’, in the case of one family ‘because we were sending him to school when he was telling us he wasn’t safe’ (LK). Others stated how it impacted on their ‘sanity, not being able to play happy families, and just being completely burnt out’ (HT). Families were also significantly affected financially, emotionally and economically. One parent recounted:

I’d given up my job. Because I couldn’t cope with my child’s school refusing. Me battling to get her ready. My second child battling. Dropping her off before school care, after school care so I could work, and she wasn’t coping. And it was just getting harder and harder. (RB)

Sometimes, leaving work meant ‘going into poverty, essentially, to make sure they had some support through education’ (TU). Parents highlighted how they were ‘more aware of how traumatic it’s been. That’s because my own mental health has started suffering’ (LF).

The emotional and financial impact on families sometimes also led to *tough decisions about school placement* (subtheme 2.4), with mothers reporting having to make decisions about whether to change schools or remove their child/ren from formal educational settings and start home-schooling. They explained that school experiences can be entirely ‘teacher driven’ (TU), so they would often consider persisting in the same school and hope for a supportive teacher in the following year. Some autistic parents constantly debated ‘the million dollar “what do I do?” question . . . do I home-school? Do I do distance education? What do I do?’ (TU). Others knew what they wanted for their children: ‘to be rich enough [that] I don’t need to work, and I can home-school my kids, with all the social interactions and activity and variety and learning they need. But that’s a pipe dream’ (AY). For others, the decision to remove their children from school was guided by consistently poor interactions with schools, including even ‘the way the principal treated him’ (LK).

### Theme 3: successful school partnerships are built on mutual respect

Although many mothers expressed significant challenges interacting with schools, some spoke of successful interactions. Those that shared positive experiences emphasised that having *educators who are flexible and open to learning is key* (subtheme 3.1) to creating successful school experiences, where ‘there’s respect for strengths, but also acknowledgment of the challenges’ (TU). Autistic mothers who had positive experiences with schools emphasised how the teacher would say, ‘‘we’re going to try this, if it doesn’t work, we’ll try another way, it’s okay’’ (GI), and this flexibility ‘would allow [child] to be able to engage’ (CO). This could be enormously reassuring: ‘even when there’s a bump in the road, you know that everything’s going to be okay’ (ML). The effects were also felt by their children: ‘he felt like he was listened to, it was an environment that enabled him to be successful’ (ST). As one parent put it: ‘honouring or respecting each individual’s particular pace of learning, pace of development and the way their mind works’ leads to a ‘safety that comes from understanding and accepting and honouring individuality’ (CO).

One autistic mother detailed a response from the school principal, who reportedly said ‘‘we will always make it work, we will always find a way’’ (ML), which had a positive impact on their relationship. This mother explained feeling ‘very lucky we found this school and this school is supporting us . . . I feel there’s always a path forward no matter what’ (ML). Autistic mothers further described how flexibility can come in many forms – in learning, teaching and settings. They were emphatic about wanting ‘to see more flexibility and creativity by schools in how they deliver education’ (DE). Many were keen to see greater flexibility in how their autistic children attended school, ranging from attending for half days, a combination of home-schooling and traditional school settings, to part-time attendance. One parent explained the benefits of this flexible approach:

Technically, he is there full-time, but we have a working arrangement with the school, that if he’s not coping, that he comes home before things escalate too much. The rationale behind that being that we want school to be a positive environment for him, so we’d rather not leave him there distressed. (NW)

Autistic parents also reported feeling connected to and included in their children’s schools, when *schools value parents’ experiential expertise* (subtheme 3.2): ‘That’s when it works, when I become part of the discussion about how my child is supported’ (TU). They felt that appreciating parents’ knowledge and expertise about their children was critical to forging successful collaborations between home and school, when ‘they [schools] actually see me as a viable expert on my child, not the helicopter mother who is annoying everyone’ (TU). Another parent agreed: ‘when it’s going well, it’s because they’ve listened to me, so then I feel like I have a say in what happens when my child is not with me’ (LF).

Parents with positive experiences further described how their children’s schools ‘rely on a lot on parent input’ (GI) and when teachers are developing plans and setting goals with families, they would discuss challenges openly and ask directly for parents’ input. As one parent reported, ‘they’ll say, “we’ve noticed his behaviour, it’s not a problem, but how would you suggest we maybe turn it around to make it a positive experience?”’ (GI). Another mother described the teachers as ‘really proactive, and also as very human, so quite understanding and warm with me as a parent. It’s more that partnership . . . and not like, “yes, I’m a teacher, therefore I know everything about teaching,” and your contribution is nothing’ (AY). Parents reported that, while ‘shaming and othering’ tends to lead them to feel ‘powerless’ (TU), these power imbalances start to break down when parents feel ‘respect and trust in our [their] expertise and experience with [child]’ (LK), and when that sense of ‘she’s your child, and you know her best’ is acknowledged (AY).

This respect and understanding from teachers also needed to extend to their children. One parent reported how the school proactively engaged parents ‘right from the start’ and the teacher ‘tried to look at things through the child’s eyes, and would also engage with me, and be like, “hey, what do you think about this idea? Would that help?”’ (AY). They described wanting their children to be cared for, to ‘*feel safe and secure, and connected’* at school (subtheme 3.3): ‘rather than being that teacher up here, child down here, you do as I say and must obey . . . [teacher] really worked with the kids and partnered with them, and that made a big difference’ (AY). Autistic parents emphasised they and their autistic children need ‘teachers that are emotionally available in a way that true connections can form’ (FZ) and who can ‘manage with compassion’ (RB).

Autistic parents wanted schools to ‘build up [children and parents’] confidence in school being a safe space’ (JR). Parents were aware of what made their children feel safe, including ‘lots of little, tiny incidental conversations that show respect. As soon as that happens, they feel safer, and the minute they feel safer you will get as much participation as you will get from them’ (ML). Another parent described how her child ‘thrives in environments with people she actually feels connections with. If she doesn’t feel connected to someone, it just does not work, and she feels really uncomfortable’ (UC). Even simply having the option to use a ‘safe place’, where the child ‘gets up, and walks around, and rolls on the floor’, and ‘if that’s what he needs, to regulate, that’s fine’ (NW). Overall, they felt that the hallmarks of a safe and caring environment at school for both students and parents alike came down to whether ‘the teacher has empathy, the teacher has flexibility, and the teacher has respect for the child and the family’ (TU).

### Theme 4: the critical role of self-advocacy

Autistic parents’ advocacy and self-advocacy were also considered essential in contributing to positive school experiences. They often described themselves as having ‘a very keen, social justice button’ (GI), which contributed towards their role as advocates ‘trying to stand up for my children’ (GI). They also described how their *confidence in advocacy and self-advocacy had grown over time* (subtheme 4.1). This growth, however, was often not easily achieved: ‘it takes a long time to learn to be your child’s best advocate’ and also to learn ‘how you can speak up’ (LF). This mother also reflected on ‘when [child] was in pre-primary, people weren’t listening to me . . . it came down to me not being confident enough to say that she needs things, or that something’s wrong, because I was constantly blamed that it was parenting’ (LF). But parents felt determined ‘to keep going’ (RB), as they felt they had no other choice. Developing a sense of what it means to be autistic often helped them to be stronger self-advocates, and advocates for their children and the autistic community more broadly: ‘there’s a confidence that comes with self-acceptance [of being autistic] and knowing that if you don’t ask, you won’t get your needs met’ (FZ). One parent described how she put this knowledge into practice:

I made a point when we went to [new school], in my initial conversations with everyone, I was being really open. Proactively telling them, [child] and I are autistic, because I want people to understand her. And the best thing I can do is to be really open about this. I’m proud of our neurodivergence. (UC)

Indeed, our participants were adamant of the need to convey to schools that ‘*there is nothing wrong with being autistic*’ (AY; subtheme 4.2): ‘they need to accept that difference is not a bad thing, it’s just difference. And if we are flexible and able to see the positive and negatives of difference, everyone will be embraced’ (ML). Autistic mothers, therefore, stressed the importance of advocating for acceptance from early on, as one parent explained:

‘once I was at my son’s kinder when [teacher] said to me “what happens to these kids? How do they grow up?” and I said “well, I’m here, look at me” . . . that disclosure enabled [teacher] to see, this is what being autistic can look like’ (PQ).

They emphasised ‘the most important skills are self-awareness and self-advocacy, the learning to know yourself and then the learning to advocate for what you need’ (ML). They also instilled these positive attitudes in their children, so they could be ‘really confident in themselves to go into the world not feeling like they’re wrong or broken or stupid or the odd ones out and to have a really positive sense of who they are’ (LK). As such, they felt their own self-advocacy could have a positive impact on their children: ‘I think my diagnosis is fabulous modelling for them about autistic positive living . . . they can see me making a difference’ (TU).

## Discussion

This study focused on autistic mothers’ experiences of interacting with schools regarding their autistic child/ren’s education. Autistic mothers painted a picture of how they were consistently made to feel like ‘the problem’ and the ‘enemy’. For the most part, these mothers did not feel listened to by schools. It was as though they were in a never-ending battle – one which resulted in significant mental health and economic difficulties that impacted the whole family, ranging from severe stress and mental health issues to financial hardship. Despite these challenges, there were also glimmers of hope with some autistic families experiencing successful school partnerships. These were built on mutual respect, openness to sharing knowledge and expertise, and problem-solving together. Being a proactive advocate for their child and for themselves, also played a critical role in promoting understanding and acceptance of being autistic within the broader school community.

Our autistic mothers’ reports of the substantial challenges engaging with schools echo previous research on non-autistic parents’ school experiences (Burke et al., 2020; Garbacz et al., 2016; Kendall & Taylor, 2016). Non-autistic parents consistently report that teachers’ lack knowledge about autism and how to respond best to their autistic children’s needs (Azad et al., 2018; Lilley, 2015), often leading to an ongoing ‘battle’ between parents and schools (Gray et al., 2023; Hurlbutt, 2011; Parsons et al., 2009). Making tough decisions about school placement was also a constant area of stress for our autistic parents – an issue that families of autistic children frequently report having to grapple with (McNerney et al., 2015).

It is not surprising that the ongoing adversarial interactions between parents and schools took an emotional toll on mothers we interviewed, including experiences of deteriorating mental health, heightened stress, and serious impacts on employment and living conditions (see also Heyworth et al., 2021; Pohl et al., 2020). While non-autistic parents of autistic children have described the substantial impact of negative school experiences on their own and their child’s mental health (Gray et al., 2023; Marsack-Topolewski & Church, 2019), such negative experiences may have a disproportionate effect on autistic parents, who have a greater likelihood of developing co-occurring mental health conditions (Uljarević et al., 2020) and may themselves be more likely to have had experienced challenging educational experiences. Given continuing reports that autistic women experience even greater health disparities and clinical/diagnostic inequities, mental health risks and exposure to difficult education experiences may be further exacerbated for our sample (Lai et al., 2023). Autistic mothers who experienced their own educational barriers, disadvantage or trauma, reported seeing these detrimental events repeated in their child’s life, which may have resulted in increased stress and distress, and, indeed, triggering of past traumas. These experiences may well be further compounded by having less access to suitable resources and supports to tackle some of these mental health and school interaction challenges in the same way that neurotypical parents might (Heyworth et al., 2021; Muniandy et al., 2021).

Notwithstanding these difficulties, autistic mothers often displayed great determination through their sustained efforts and advocacy. They demonstrated their commitment both to their child and to educating schools and teachers about neurodiversity and autistic pride. For some, this determination and willingness to develop strong relationships with their children’s schools and teachers resulted in positive interactions. However, this was not without the extra effort often required by autistic parents to communicate with teachers and schools and advocate for their child’s needs and their own. Parents felt they had a lot to offer teachers as autistic people themselves, including an informed and intuitive understanding about their child’s needs, interests and preferences. They emphasised that positive partnerships between home and schools are built on reciprocity, sharing of knowledge, experiential expertise and problem-solving together.

These findings echo existing findings with non-autistic parents, highlighting the need for a nurturing educational environment with open communication and an active partnership between home and school (Azad & Mandell, 2016; Azad et al., 2018; Brede et al., 2017; McNerney et al., 2015). Why, then, are such positive partnerships so often not achieved? Broader systemic factors – including inadequate funding and training to support diverse educational needs – might partly explain the challenge. Funding pressures, for example, can place significant constraints on school leaders and teachers’ capacity to adopt the flexible approach that autistic parents so desperately wanted. This can in turn affect staff morale, and staff attitudes towards inclusion. Indeed, one UK-based study showed that teachers held more negative attitudes towards the inclusion of their autistic students when they believed they had insufficient resources to facilitate such inclusion, than teachers who did not hold such beliefs (Leonard & Smyth, 2022). It is plausible that such negative attitudes could influence the quality of home–school relationships and exacerbate adversarial interactions.

Previous research has focused on the more proximal factor of communication issues as a major concern for both teachers and non-autistic parents, highlighting that ideal parent–teacher interactions start with shared understanding (Azad & Mandell, 2016; Azad et al., 2018). The ‘double empathy problem’ (Milton, 2012) reveals, however, why effective parent–teacher partnerships might not be straightforward to develop and maintain for autistic parents specifically. It is well-established that non-autistic people can struggle to make sense of autistic people’s thoughts, feelings and behaviours (Davis & Crompton, 2021; Milton, 2012; Mitchell et al., 2021), and that this misalignment between the minds of autistic and non-autistic people can result in a breakdown in reciprocity and mutual understanding. This perspective emphasises that effective communication is shaped by both parties and that extra attention is needed to enhance mutual understanding between autistic parents and non-autistic educators.

Autistic parents also wanted teachers to be responsive to, and accepting of, their autistic students’ individual strengths, interests and needs and to develop caring relationships with them. These sentiments are consistent with a relational or ethics-of-care-based view of education, which has long suggested that care is an essential aspect and aim of education, with teachers developing caring relationships with their students, and students reciprocating this (Noddings, 1984, 2012). This relational approach to education is especially important for autistic children, who often report negative experiences of school (see Horgan et al., 2023, for review) – but value it enormously when care is shown, and their views, needs, preferences (Gray et al., 2023), strengths and interests are taken seriously. Establishing care and connection in schools has the potential to provide autistic pupils with the respect and agency they require to grow and lead flourishing lives (Pellicano & Heyworth, 2023).

### Limitations

This study had several limitations. First, our findings may not be representative of the autistic population as our autistic parents all identified as female, were mainly well-educated (i.e. attained a university degree) and were mostly of white racial/ethnic background. Second, our sample was self-selecting, rendering it possible that some of those motivated to participate already had challenging experiences with their child’s school(s). Third, the majority of autistic mothers who participated had autistic children who were mostly able to articulate their experiences using verbal communication. As a result, the challenges and experiences shared may well be an underestimate of those experienced by families of autistic children, who may also be non-speaking or minimally speaking, have additional intellectual disability, require higher levels of support and/or are from more diverse backgrounds (both culturally and socially).

## Conclusion

Autistic mothers frequently reported feeling like the enemy during their interactions with teachers and schools, and constantly felt blamed for their autistic children’s challenges. They were adamant that ‘it doesn’t have to be like this’ – that strong, trusting and collaborative connections between teachers, autistic parents and their autistic students should not be out of reach. Some autistic mothers’ positive experiences with schools demonstrated that such connections are entirely possible. Future research should examine how best to integrate autistic parents’ experiential expertise in schools, and create safe learning environments that foster respectful and meaningful connections between students, teachers and parents.

## Supplemental Material

sj-docx-1-aut-10.1177_13623613241297223 – Supplemental material for ‘I’m sick of being the problem’: Autistic mothers’ experiences of interacting with schools for their autistic children Supplemental material, sj-docx-1-aut-10.1177_13623613241297223 for ‘I’m sick of being the problem’: Autistic mothers’ experiences of interacting with schools for their autistic children by Aspasia Stacey Rabba, Jodie Smith, Gabrielle Hall, Vanessa Alexander, Kate Batty, Poulomee Datta, Emma Goodall, Melanie Heyworth, Siobhan Lamb, Wenn Lawson, Rozanna Lilley, Katy Reid, Najeeba Syeda and Elizabeth Pellicano in Autism

## Notes

1. It is entirely possible that autistic parents were included in existing studies but information on parent diagnosis (or identity) was rarely collected or reported in such studies – which means we cannot glean the experiences of autistic parents specifically.

2. Despite efforts to recruit both autistic mothers and fathers, we received no expressions of interest from autistic fathers. The fact that only mothers responded suggests that gendered societal expectations of maternal responsibility for schooling are also found among autistic parents.
