# Supplementary material for: ‘I’m sick of being the problem’: Autistic mothers’ experiences of interacting with schools for their autistic children
Source: Autism. 2024 Nov 24;29(4):1034–46. doi: 10.1177/13623613241297223 (PMC11967083; doi:10.1177/13623613241297223)
Supplement: sj-docx-1-aut-10.1177_13623613241297223 – Supplemental material for ‘I’m sick of being the problem’: Autistic mothers’ experiences of interacting with schools for their autistic children [file sj-docx-1-aut-10.1177_13623613241297223.docx]

**Start the Interview**

**INTRODUCTIONS**

**To start, please tell me a bit about you and your family at home.**

- *How many children do you have? How many are autistic? How old are they?*
- *What type of school do they go to? What grade are they in?*
- *Besides you and your children, who else lives with you at home?*

**QUESTIONS**

**1 YOUR EXPERIENCE OF SCHOOL(S)**

**1a) Can you tell me a bit about your experience of your child/ren’s school(s)? [Do they go to the same school?]**

**1b) How much do you know about what life is like at school for your child/ren? How did you find out this information?**

**1c) Are there things you don’t know about your child/ren’s life at school that you’d like to know? If so, what are they? And how might you find out these things?**

**Prompts:**

- *Do you remember your first contact/communication with early learning or primary schools? What was it like?*
- *What made you choose the school you have chosen for your child/ren?*
- *How have transitions gone in general [between schools, or within schools]?*
- *Has your child/ren always been at this school? If not, which school did they used to go to? Why did your child leave that school?*
- *As your child has developed/as you learn more, has your experience of your child/ren’s schooling changed over time?*

**2 COMMUNICATION AND INTERACTIONS BETWEEN SCHOOL AND HOME**

**Now I’d like to move on to talking more specifically about how you communicate and interact with your child/ren’s school.**

**2a) Can you tell me about interactions you have with teachers or other professionals at your child/ren’s school? Is this something you would like more/less of?**

**Prompts:**

- *What are some reasons why you usually communicate with your child’s school?*
- *Who do you usually communicate with?*
- *How do you usually communicate (i.e., impromptu face to face chat, email letters, meetings)?*
- *Have you felt able to support your child/ren’s learning or social experience at school?*
- *Would you like to participate more in your child/ren’s learning or social experience at school?*

**2b) Could you give me an example of a time when things worked really well between you, your child/ren and the school?**

**2c)** **What about a time when it didn't work so well with school?**

- Do you know why this experience may have been different from the first example?
- Do you think there is anything that you or the school could have done differently to improve this experience?

**2d) Are there any strategies shared between home and school? If yes, how?**

**Prompts:**

- *Has this been successful in supporting your child? If yes, in what way? If no, why?*
- *What’s your opinion of homework? Is it difficult or easy to get your child to do homework? Do you or your child’s teacher modify homework? If so, how?*

**2e) What do you think would promote a positive working relationship with your child/ren’s school?**

**Prompts:**

- *What would help to increase your involvement with your child/ren’s learning or social experience?*
- *What is your relationship with your child/ren’s wider school community like?*

**2f) Communication between homes and schools *may* look a bit different since the COVID pandemic. Do you feel it is different? If yes, how so?**

**BREAK [if required]**

**3 PARENT INVOLVEMENT**

**3a) Parents may be involved in their child’s education in different ways, at home and at school. Can you tell me about your involvement in your child’s education?**

**Prompts:**

- *Has your support changed over time?*
- *Has the school been open to having an external person (e.g., family member, support worker, therapist) coming into the school to support you and your child?*
- What do you think about Individual Education Plans/Individual Learning Plans? Does your child have one? Do you feel they help? In what ways? If not, why not?

**3b) How involved are you in decisions made about your child’s learning at school? Are you satisfied with this?**

**3c) Do you take part in any of your child/ren’s activities at school** [if need examples, i.e., attend any meetings, volunteer, policies, attend school trips, etc.]**?**

**Prompts:**

- *How about any learning activities at home [if need examples, i.e., help with homework, read/play games with child, etc.]?*
- *Are you satisfied with how much you are involved with your child/ren’s school?*

**4 INCLUSION/BELONGING**

**4a) Have you disclosed that you are autistic to teachers/school staff? Why/why not?**

**Prompts:**

- *What prompted you to share/not share this?*
- *How was this received?*
- *Did this have an impact on your subsequent interactions?*
- *Have any accommodations or supports been offered to you to assist with your relationship between home and school?*
- *What would you like to have been offered?*
- *What do you think could be done to help partnerships between autistic parents and schools?*
- *Has your child’s school(s) provided inclusive/helpful information for parents (e.g., large print, extra meeting time, transgender, same-sex couples, etc.)?*

**4b) People have different ideas about autism. How do you think the school understands autism – and other forms of disability/neurodivergence? Does this match your understanding?**

**Prompts:**

- *Does the school recognise your child/ren’s strengths? If so, how?*
- *Has the school changed your ideas about autism? Or vice versa? -What have they done to support you in that way? Or what have YOU done to support their understanding?*

**4c) Do you think your diagnoses have made a difference to you and your child’s experiences of school, family, or yourselves? In what way?**

**5 YOUR IDEAL PARTNERSHIP BETWEEN HOME AND SCHOOL**

**5a) We’re up to the final question! In an ideal world, what would the relationship between home and school look like for you?**

**Prompts:**

- *How would your ideal school support your child and your family as a whole?*
- *How would your ideal school understand and accommodate neurodiversity, including autistic parents?*
- *How do you think schools could learn more about how to support all families?*
- *What kind of values would your ideal school community have?*

**ANY OTHER ISSUES**

**Are there any issues that we haven’t covered that you think are important?**

**Next steps**

That’s all my questions. Thanks so much for speaking with me. Once we have finished seeing all the young people and speaking to parents and school staff, we will collate the data and try pull it all together. At the end of the project, we will send you a report describing what the project was about, what we did, what we found and what it all means. In the meantime, if you have any further questions or would like to contact me for anything else, here are my contact details.

**End of interview**
